# Mechanisms of Nuclear Export in Cancer and Resistance to Chemotherapy

**DOI:** 10.3390/cancers8030035

**Published:** 2016-03-14

**Authors:** Mohamed El-Tanani, El-Habib Dakir, Bethany Raynor, Richard Morgan

**Affiliations:** Institute of Cancer Therapeutics, University of Bradford, Bradford, West Yorkshire BD7 1DP, UK; E.H.Dakir@bradford.ac.uk (E.-H.D.); bethrayy@hotmail.com (B.R.); R.Morgan3@bradford.ac.uk (R.M.)

**Keywords:** Nuclear export, CRM1, Ran, nucleocytoplasmic transport

## Abstract

Tumour suppressor proteins, such as p53, BRCA1, and ABC, play key roles in preventing the development of a malignant phenotype, but those that function as transcriptional regulators need to enter the nucleus in order to function. The export of proteins between the nucleus and cytoplasm is complex. It occurs through nuclear pores and exported proteins need a nuclear export signal (NES) to bind to nuclear exportin proteins, including CRM1 (Chromosomal Region Maintenance protein 1), and the energy for this process is provided by the RanGTP/RanGDP gradient. Due to the loss of DNA repair and cell cycle checkpoints, drug resistance is a major problem in cancer treatment, and often an initially successful treatment will fail due to the development of resistance. An important mechanism underlying resistance is nuclear export, and a number of strategies that can prevent nuclear export may reverse resistance. Examples include inhibitors of CRM1, antibodies to the nuclear export signal, and alteration of nuclear pore structure. Each of these are considered in this review.

## 1. Introduction

**Figure 1:**
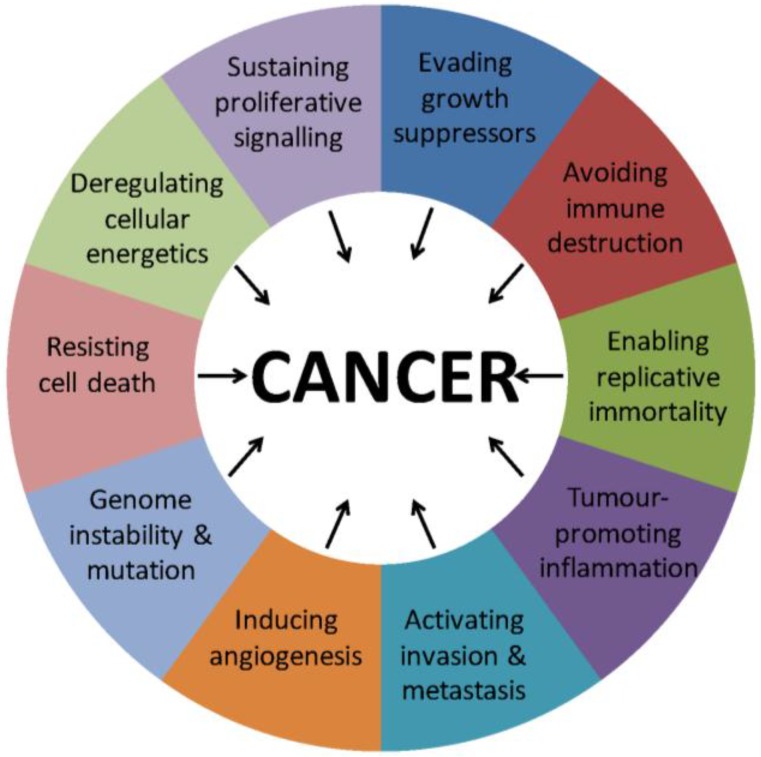
The Hallmarks of Cancer. This figure displays the hallmark features acquired by most cancers. These characteristics contribute towards the pathogenesis of cancer and may arise due to mutations.

Cancer is a disease characterised by genetic mutations leading to uncontrollable cell division and invasion into local tissue, which eventually spreads into distant tissue, a process known as metastasis. Cancer is a very heterogeneous disease, although it has been proposed and widely accepted that there are hallmark features common to most cancer cells (Figure 1) [1], the development of which is driven by mutations. Mutations are acquired through either exogenous or endogenous DNA damage. In response to DNA damage, the cell can activate DNA repair systems and enter cell cycle arrest in order to limit further damage. A key component of this response is the p53 tumour suppressor gene that controls cell cycle arrest, and which can also, if catastrophic damage occurs, induce apoptosis (*i.e.*, programmed cell death) [2]. These mechanisms prevent mutations from being passed onto future generations of cells. As such, the rate of spontaneous mutations occurring in normal tissue is low, although its exact incidence is difficult to measure [3]. The acquisition of mutations may be viewed as a stepwise process and often there is a failure of DNA repair mechanisms. When these mechanisms are disrupted, the cancer cells can acquire mutations at a rapid rate and acquire hallmark features. This can be seen as a process of Darwinian natural selection, as a heterogeneous cancer cell population includes cells that may become dominant due to favourable mutations [4]. An alternative theory to the acquisition of mutations is that cancer cells revert to an ancient survival programme under stress, which prioritizes the continuation of individual cellular life over that of the multicellular organism [5]. This article focuses on drug resistance, which can be considered a result of the selection pressure on cancer cells when exposed to chemotherapy [1]. There appears to be redundancy in many signalling pathways, which the chemotherapeutic drugs target. When one pathway is shut off, there may be residual function in another pathway, and this could be further enhanced by additional mutations [6]. Drug resistance in cancer cells is comparable to antibiotic resistance in the sense that there are slow growing cancer stem cells that possess tumour initiation and self-renewal ability, and can escape the effects of drug treatment by becoming quiescent, and are thus comparable to populations of persister cells in microbial biofilms [7]. This article will focus on the mechanisms of drug resistance, particularly with respect to nucleocytoplasmic transport, and potential targets in overcoming resistance. Many tumour suppressor proteins and negative regulators of the cell cycle need to be in the nucleus to regulate gene expression and promote apoptosis. Nuclear transport has been an intensive area of research and the underlying mechanisms provide possible molecular targets for therapeutic intervention. This review considers the structures and mechanisms involved in nuclear export, and how the inhibition of nuclear export may be able to reverse drug resistance.

## 2. Nuclear Export Structures and Mechanisms

There are a number of structures involved in the nuclear export of proteins larger than 40 kDa including the nuclear pore complex, nuclear export signals present on the protein to be exported, karyopherins, and Ran. Each of these will be discussed in this section.

### 2.1. The Nuclear Pore and Nuclear Envelope

**Figure 2:**
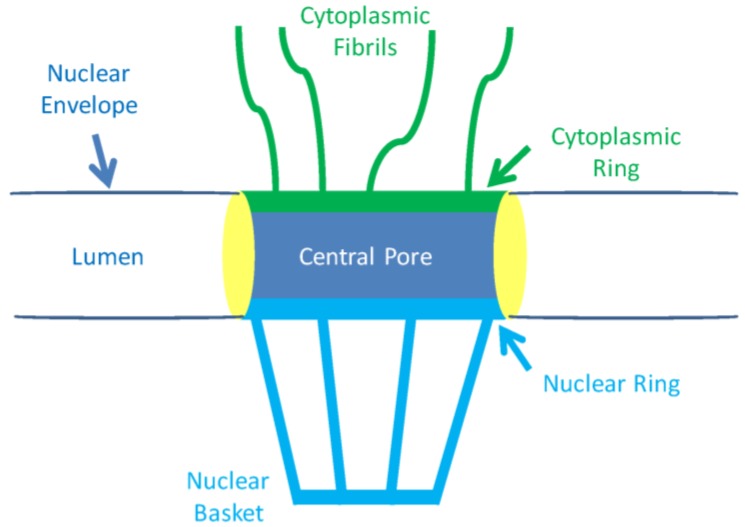
The Nuclear Pore Complex Structure. A schematic cross-section of the nuclear pore complex. The entry and exit of macromolecules from the nucleus is regulated by this complex.

A key feature of eukaryote cells is compartmentalisation; organelles are separated from the cytoplasm by membranes. Molecules can move between these compartments through specialised transport pores. The nuclear envelope [8] is composed of an inner and outer membrane, it prevents various macromolecules, including proteins, from freely moving into the nucleus from the cytoplasm. The nuclear envelope membranes are joined intermittently by nuclear pore complexes, the specialised transport pores of the nucleus. These nuclear pores control the entry and exit of macromolecules from the nucleus [9], and allow small molecules and ions to freely diffuse through, but not larger molecules such as proteins and ribosomal subunits. Larger molecules instead move through the nuclear pores in a selective, active process facilitated by a number of mobile transport receptors. Nuclear pores are formed by a structure known as the nuclear pore complex that has a total molecular mass of 125 mDa and contains up to 100 proteins known as nucleoporins (Nups). Nuclear pores have a symmetrical structure with filaments on both sides, and those on the nuclear side form a basket [10] (Figure 2). There are a variety of Nups; transmembrane Nups that anchor the nuclear pore complex into the nuclear envelope, structural Nups, and FG Nups that contain repeating amino acid sequences composed of phenyalanine (F) and glycine (G). FG Nups have no secondary protein structure and form filaments, they are localised to the central nuclear pore region of the nuclear pore complex, and extend to filaments on its cytoplasmic and nucleoplasmic surface [11]. FG Nups, for example NUP 62 that is located on the cytoplasmic side of the nuclear membrane, have been shown to be involved in transport across the pore.

### 2.2. Nuclear Localisation and Export Signals

Nuclear import and export are governed by specific amino acid sequences in the target protein [12]. Sequences involved with import into the nucleus are called nuclear localisation signals (NLS), whilst those involved with export out of the nucleus are called nuclear export signals (NES). There are two types of NLSs, those consisting of a single part up to eight amino acids long and those consisting of two distinct parts separated by a segment of 10 amino acids [13]. NESs consist of a hydrophobic sequence characterised by a leucine-rich motif of amino acids. The typical spacing is L_AAA_L_AA_L_A_L where L is leucine and A is any other amino acid [14].

### 2.3. Karyopherins

Proteins moving in or out of the nucleus must do so through the nuclear pore. Proteins larger than 40 kDa cannot pass through via passive diffusion, but instead depend upon an active process facilitated by a family of transport receptor molecules known as karyopherins [15]. Karyopherins are mobile receptors that interact with Nups to allow the movement of proteins through the nuclear pore, and can be divided into importins and exportins that are, respectively, involved in importing and exporting proteins. There are also transportins that can move in both directions. Nineteen karyopherins have been identified to date. Chromosome maintenance protein 1 (CRM1) is the primary exportin receptor. NESs bind to CRM1 with low affinity and when NESs that bind with high affinity were over expressed *in vitro*, the CRM1-NES complex became trapped in the filaments of the nuclear pore on the cytoplasmic side. A possible explanation for the low affinity of the NES is that it enables CRM1-NES export complex disassembly [16].

### 2.4. RanGTP

**Figure 3:**
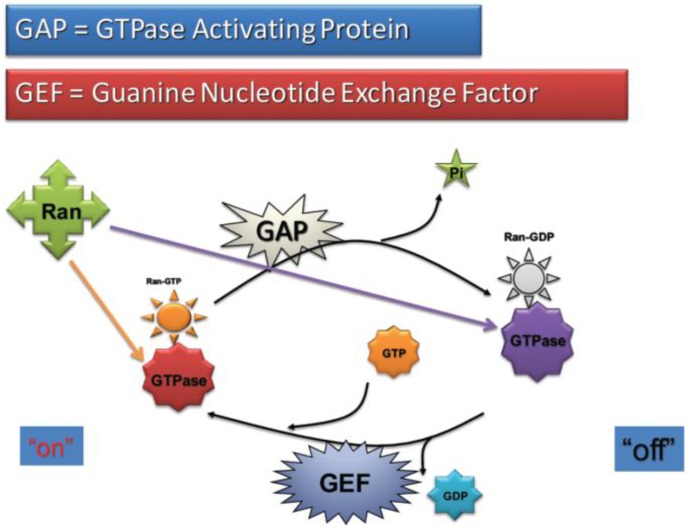
Regulation of RanGTP/GDP Switching. This diagram shows the cycling of Ran between the active GTP state and inactive GDP state. This cycling is regulated by GTPase activating proteins (GAPs) and guanine nucleotide exchange factors (GEFs).

**Figure 4:**
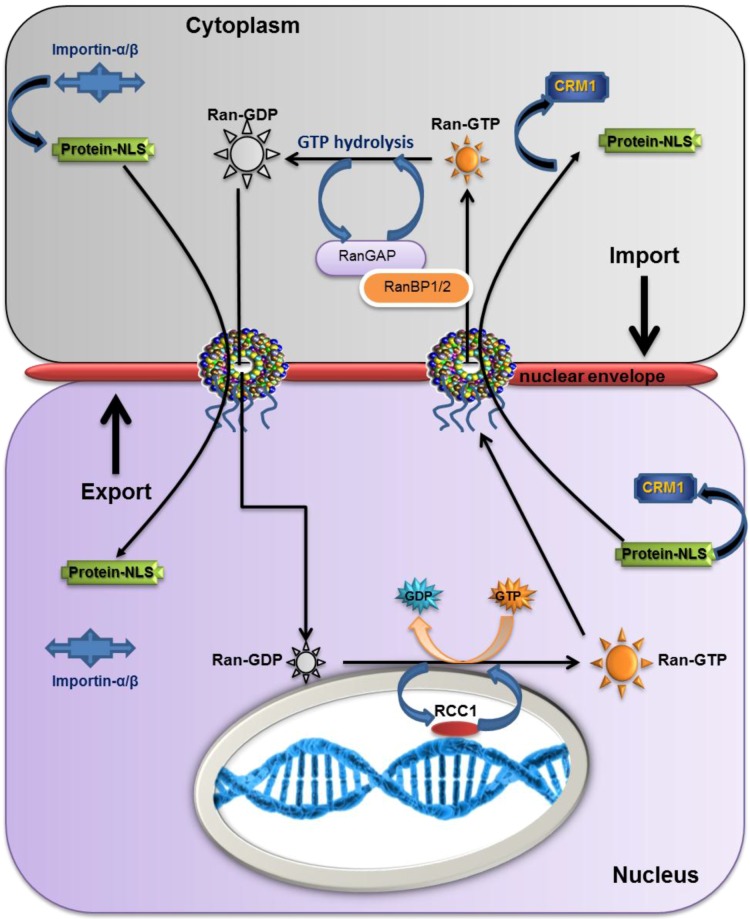
RanGTP Signalling. This diagram shows the overall mechanism of nuclear import and export. Ran assists in both of these processes by forming and dissociating complexes. This is also enabled by regulatory proteins RCC1, RanGAP, and RanBP1/2.

The movement facilitated by the karyopherins is an active process that requires energy. This energy is supplied by the RanGTP binding proteins, which act as molecular switches that move between an active GTP-bound state and an inactive GDP-bound state. Two processes are involved in GTP–GDP cycling, hydrolysis and dissociation. In hydrolysis, there is destruction of a phosphate bond: GTP→GDP + Pi (phosphate). Dissociation causes the release of GDP from Ran and a free GTP will then take its place if there are high levels of GTP in the cytoplasm. Ran possesses both intrinsic hydrolysis and dissociation activities, although each occurs at a very low rate. There are two groups of regulators that assist Ran in switching between the GTP- and GDP-bound states. The GTPase-activating proteins (GAPs) cause a change in Ran conformation, increasing the rate of intrinsic hydrolysis of RanGTP to the inactive GDP state (Figure 3). GTP hydrolysis is further stimulated by Ran binding proteins 1 and 2 (RanBP1 and RanBP2) [17]. Conversely, guanine nucleotide exchange factors (GEFs) bind to RanGDP and catalyse the exchange to RanGTP. Hydrolysis and dissociation occur at an equal rate (Figure 3). RCC1 (regulator of chromosome condensation) is the GEF for Ran, and is also involved in controlling mitosis, as such it is bound to chromatin and is located within the nucleus [18]. RanGAP and RanBPs are located in the cytoplasm, resulting in a concentration gradient with a higher concentration of RanGTP in the nucleus and a higher concentration of RanGDP in the cytoplasm [19] (Figure 4).

### 2.5. The Overall Export/Import Mechanism

The import mechanism begins with the formation of a complex in the cytoplasm between importins such as importin-α/β and cargo proteins that have a nuclear localisation signal (NLS). Once this complex has been formed karyopherins attach to the nuclear pore complex through interactions with nucleoporins. When the complex reaches the nucleus, RanGTP causes the complex to disassemble by binding to importin-β. The protein is then released into the nucleus, while the importin-RanGTP moves into the cytoplasm through the nuclear pore. The converse is true for export where a protein with a NES within the nucleus binds to an exportin and this in turn binds to RanGTP to form a NES-exportin-RanGTP complex. The complex then translocates through the nuclear pore to the cytoplasm. There is hydrolysis of the RanGTP to GDP and the complex dissociates releasing the protein into the cytoplasm. Afterwards, Nup88 recruits the cytoplasmic CRM1 to nuclear pore filaments that promote the recycling of CRM1 back to the nucleus so it can take part in export. This recycling occurs in a temperature and Ran-independent manner [20,21,22,23].

## 3. The Role of Nuclear Export in Cancer and Drug Resistance

Excessive nuclear export may be a factor in the development of cancer and resistance to chemotherapy. Consequently, inhibition of nuclear export is a potential area for treatment and reversion of resistance. Modification of nuclear export can occur in a number of ways, principally through changes to CRM1, the NES, and nuclear pore architecture. In this section each of these is looked at in turn.

### 3.1. CRM1 and Cancer

CRM1 is involved in the export of drug targets, tumour suppressors, and cell cycle inhibitors from the nucleus to the cytoplasm and these CRM1-mediated events have been implicated in breast cancer. BRAD1 (BRCA1-associated RING domain protein 1) has tumour suppressor features, and is exported to the cytoplasm where it can exert pro-apoptotic effects. BRAD1 interacts with BRCA1 (breast cancer type 1 susceptibility protein) and reduces the ability of the latter. Excessive export of BRCA1 via CRM1 from the nucleus to the cytoplasm can lead to the development of cancer [24]. CRM1 mediated export has also been implicated in colon cancer through its interaction with the APC tumour suppressor, in leukaemia via the BCR-ABL tumour suppressor, and export of p53 into the cytoplasm in a variety of cancers [25,26,27].

### 3.2. CRM1 Inhibitors

Leptomycin B, a secondary metabolite of *streptomyces* bacteria, was the first CRM1 inhibitor to be identified. It alkylates a reactive cysteine residue (cysteine 528) on CRM1, preventing it from binding to the NES, and thus blocks export by preventing formation of the CRM1-NES-RanGTP complex. Leptomycin B has been demonstrated to have efficacy *in vitro* and in animal models, although when introduced to humans in a phase 1 clinical trial there was dose limiting toxicity with severe malaise and anorexia [28]. A number of other CRM1 inhibitors have been developed, all of which act by inactivating cysteine 528, examples include ratjadone analogs, synthetic leptomycin B derivatives and selective inhibitors of nuclear export (SINE).

## 4. Application of CRM1 Inhibitors

### 4.1. Topoisomerase

During DNA transcription and replication, DNA becomes tangled due to its helical structure. Topoisomerase unwinds DNA allowing the supercoiled structure to relax by temporarily inducing strand breaks, through a process known as transesterification. Transesterification involves the formation of a covalent bond between the tyrosol oxygen on the topoisomerase and a phosphate on the backbone of the DNA, breaking the phosphodiester bond [29]. Afterwards, a reverse transesterification reaction occurs; oxygen exposed on the original DNA molecule reacts with the phosphate that becomes covalently bound to the topoisomerase. This breaks the bond between the phosphate and topisomerase and restores the phosphodieseter bond within the DNA molecule [29]. Topoisomerases are divided into type I and type II. Type I topoisomerases cause single strand breaks while type II cause double stranded breaks. Topoisomerases are essential for cell survival and without them DNA replication and transcription cannot occur. They are highly expressed in proliferating cells and, as such, they present an ideal target for chemotherapy. Naturally-occurring topoisomerase inhibitors target type II topoisomerase α and cause arrest during the tranesterification stage leading to permanent double stranded breaks and cell death. One such inhibitor is etoposide, which is used to treat lymphoma, myelomas and lung cancer. However, resistance after a period of successful treatment is a common outcome, frequently due to export of topoisomerase from the nucleus to the cytoplasm. When in the cytoplasm topoisomerase II α cannot interact with DNA and so permanent double stranded breaks are not formed. Alternatively, the cytoplasmic topoisomerase II may act as a buffer, preventing etoposide from reaching the nucleus and thus resulting in resistance [30]. *In vitro* studies of drug resistant myeloma cells exposed to CRM1 inhibitors, such as ratjadone, have shown that they can be re-sensitised to etoposide [31].

### 4.2. Galectin-3

Galectin-3 (gal-3), a 30 kDa protein without enzymatic activity, is a member of the β-galactoside-specific lectin family. Gal-3 exhibits pleiotropic biological functions, especially in tumours. It has roles in cell growth, apoptosis, adhesion, tumour angiogenesis, metastasis, cancer-matrix interactions and drug resistance [32,33]. Gal-3 is found in different compartments in different cells types including the nucleus, cytoplasm and on the cell surface. Its function is dictated by its location, as nuclear, but not cytoplasmic galectin-3 can induce apoptosis [31]. It was shown that in cancer cells exposed to the cytotoxic anticancer drug cisplatin, gal-3 was exported from the nucleus to the cytoplasm and as a result apoptosis did not occur. In the same study when leptomycin B was added to these cells galectin-3 could not be exported with CRM1 so it stayed in the nucleus, and apoptosis occurred after exposure to cisplatin. A clinically acceptable CRM1 inhibitor could be used to complement a variety of existing chemotherapies. However, the role of gal-3 in apoptosis has not been fully characterised, and there is increased expression in breast cancer cells that makes them more resilient to apoptosis, although both over and under-expression in macrophages leads to an increased rate of apoptosis [34,35]. Recent studies have revealed that gal-3 is up-regulated in Philadelphia chromosome-positive (Ph+) chronic myeloid leukaemia (CML) and in precursor B-lineage acute lymphoblastic leukaemia (ALL) after conditioning with bone marrow (BM) stromal cells [36,37]. Cheng and colleagues [38] reported that, in patients with acute myeloid leukaemia (AML), higher bone marrow *LGALS3* (gal-3) gene expression was a prognostic factor for shorter overall survival. Gal-3 was recently reported to be associated with an increase in drug resistance in chronic myeloid leukaemia (CML) mediated by the bone marrow microenvironment [36]. Hu *et al.* (2015) [39] found that gal-3 was dramatically up-regulated in human bone marrow-derived mesenchymal stromal cells (hBM-MSC)-conditioned acute leukaemia cell lines, and that this was associated with the activation of β-catenin signalling. Both gal-3 and β-catenin signalling were shown to be essential for the survival of acute leukaemia cells (ALCs) after they were treated with cytotoxic drugs. The same group showed that gal-3 modulated β-catenin signalling by regulating GSK-3β phosphorylation and the PI3K/Akt pathway in hBM-MSC-conditioned ALCs [39].

### 4.3. Selective Inhibitors of Nuclear Export (SINE)

Recently, a novel class of small molecule Selective Inhibitors of Nuclear Export (SINE) have been developed, which bind specifically to the NES-binding groove of XPO1/CRM1 and prevent the interaction with its cargo proteins [40,41,42]. These compounds showed anti-cancer activity in a number of solid and haematological malignancies both *in vitro* and *in vivo* [40,41,43,44,45,46,47,48,49,50]. Selinexor (KPT-330), a first-in-class selective inhibitor of nuclear export (SINE), has been developed by Karyopharma Therapeutics and is the most advanced pharmacological agent currently being evaluated in phase I/II human clinical trials for haematological and solid tumours. SINE compounds have been shown to selectively suppress tumour cells and spare normal cells in pre-clinical and clinical settings, both alone and in combination with other drugs, and thus represent a novel therapy for patients at high risk of metastasis and a novel class of anti-cancer agents [51,52,53,54].

## *5. NES* Inhibitors

Survivin is a member of the inhibitors of apoptosis family and is involved in cell division through its interactions with mitotic spindles. It is highly expressed in tumour and embryonic cells but not in normal tissue. Increased expression levels correlate with tumour grade, resistance to chemotherapy and overall survival. This makes it an ideal candidate for chemotherapy [55]. Survivin is exported from the nucleus in a CRM1 dependent manner and only exerts an anti-apoptotic affect when present in the cytoplasm. Evidence for this comes from *in vivo* experiments and the identification of nuclear survivin in colorectal cancer specimens. Higher concentrations of nuclear survivin compared to cytoplasmic survivin were associated with prolonged survival [56]. In the same study antibodies were produced against the survivin NES preventing its nuclear export, resulting in increased apoptosis and reduced resistance to chemotherapeutic agents such as cisplatin. Targeting NES to prevent nuclear export in general provides a more specific target than CRM1 and since survivin is not expressed in normal cells it is, in theory, less likely to produce side effects in patients compared to CRM1 inhibitors.

## 6. Nup Dormancy and Resistance

During cisplatin treatment of ovarian cancer cells, it was shown that some cells gain resistance to treatment and enter a transient state of dormancy. Some dormant cells then resume proliferation and eventually lose their resistance to cisplatin treatment. One study has shown that a possible cause of this transient increase in resistance and reduction in proliferation is the modification of a Nup protein. Nup62 is present on the nuclear side of the nuclear pore complex. It has FG regions and thus is involved in transport through the nuclear pore [57]. Small interfering RNA was used to knock down Nup62 expression, leading to a reversible inhibition of growth [57]. However, when Nup62 expression was knocked down in combination with cisplatin treatment, the ovarian cells gained resistance to treatment and entered a state of dormancy. Afterwards, when transient Nup62 expression was induced, the cells left their state of dormancy and proliferated rapidly. A potential treatment for these dormant cells may be gene therapy to replace the mutated Nup62 gene.

## 7. Sgk1

It has been shown that serum- and glucocorticoid-regulated kinase (Sgk1) regulates RanBP1 transcription. RanBP1 is a major effector of Ran and could potentially be a target for cancer therapy [58]. Talarico *et al.* (2015) demonstrated that a selective Sgk1 inhibitor named SI113 blocked cell cycle progression in two human hepatocarcinoma (HCC) cell lines, HepG2 and HuH-7. They consider that Sgk1 overexpression may have a central role in cancer due to its ability to regulate the Ran/RanBP1 pathway. SI113 targets and inhibits Sgk1 which turns off several pathways, including Ran/RanBP1, leading to tumour growth arrest and the induction of apoptosis and necrosis. This compound appears to have a good therapeutic index as there were only minimal signs of toxicity *in vitro* or in SI113-treated mice [59].

## 8. Conclusions

Drug resistance is a growing problem in oncology, due to the continued accumulation of mutations by cancer cells. Nuclear export is a potential area from which resistance can arise. The structure of the nuclear pore complex has been elucidated and the mechanisms involved in the export of proteins have been well documented. However, there are gaps in our knowledge of this area, especially with regard to the structural characteristics of FG nucleoporins. Drug resistance via nuclear export can only be understood with an appreciation of the structures involved. The inhibition of CRM1 has been the focus of most strategies to overcome drug resistance, although leptomycin B treatment has been disappointing due to toxic side effects on patients. Alternative CRM1 inhibitors have been developed which are superior *in vitro*. SINE inhibitors are currently undergoing clinical evaluation and results from these trials seem promising. Inhibition of the NES using antibodies is a promising area as it is a more specific treatment, however little work has been done in this area.
